# Optimization of Laser-MAG Hybrid Welding Parameters of Ship Steel Based on Response Surface Methodology

**DOI:** 10.3390/ma15124328

**Published:** 2022-06-18

**Authors:** Hongwei Sun, Jialei Zhu, Benshun Zhang, Chao Liu, Chunyu Miao, Kai Wang, Xiaoxin Zhao

**Affiliations:** 1 Jiangsu Automation Research Institute, Lianyungang 222006, China; besenzhang@163.com (B.Z.); liuchao2359@163.com (C.L.); 2 Beijing Institute of Petrochemical Technology, Beijing 102617, China; mcy242614@163.com (C.M.); zhaoxiaoxin@bipt.edu.cn (X.Z.)

**Keywords:** 10CrNi3MoV ship steel, laser-MAG hybrid welding, Response Surface Methodology, regression model

## Abstract

In this paper, the optimization of laser-MAG hybrid welding parameters of 10CrNi3MoV ship steel was developed. Using the Box-Behnken Design (BBD) model in Response Surface Methodology (RSM) and taking laser power, welding speed and welding current as response factors, the design matrix was completed and verified by experiment. The regression model associated with welding parameters was established by measuring the response indices, such as penetration, tensile strength and impact absorption energy. Through the model check, it was found that the accuracy of penetration and tensile strength of the model was high, and the optimized parameters were as follows: laser power (P) = 3700 W, welding speed (V) = 0.8 m/min, wire feeding speed (Vs) = 7 m/min. On the premise of meeting mechanical performance inspection standards, the maximum penetration was 8 mm.

## 1. Introduction

**Figure 1:**
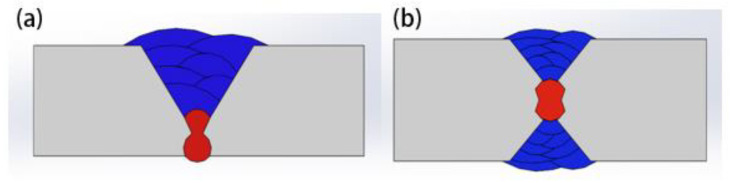
Schematic diagram of X and Y groove (**a**) Y-groove (**b**) X-groove.

In recent years, China’s shipbuilding industry has developed very rapidly, and welding technology plays a great role in the field of shipbuilding. The steel used in shipbuilding is mostly medium thick plate. Generally, Y-shaped and X-shaped grooves are selected for welding by the process of backing welding, multi-layer and multi-pass filling. The welding grooves are shown in Figure 1.

It can be seen from Figure 1 that the penetration and quality of the backing weld determine the dimension of the groove, and the groove will directly affect the residual stress distribution. If the groove dimension is inappropriate, problems can easily arise, such as lack of fusion, joint embrittlement and welding deformation. Rectification and repair after welding will affect the construction progress and increase the cost. At present, traditional arc welding technologies, such as SMAW, SAW and TIG/MIG, are commonly used for backing welding in shipbuilding, which have some disadvantages, such as poor penetration ability, low production efficiency, wide heat affected zone, large residual stress and large deformation. Kang et al. studied the thermal cycle effect of 10CrNi3MoV steel welded by the traditional welding method. They found it not only had low welding efficiency, but also had the defect of large residual stress [1]. In order to improve welding efficiency and quality, it is necessary to introduce new high-quality and efficient welding technologies.

In recent years, laser-arc hybrid welding technology has attracted extensive attention in the field of ship manufacturing. It overcomes the shortcomings of laser welding and arc welding single processes and can produce large penetration under low heat input. The use of laser-arc hybrid welding for backing welding of ship steel can make full use of the advantages of hybrid welding to optimize the groove dimensions, improve production efficiency and weld performance [2].

Laser-arc hybrid welding technology involves many process factors, and the cost of the traditional optimization process is high. The newly developing response surface method (RSM) can establish the model between the controllable welding process parameters and the expected response value, and has the advantages of economy and efficiency in the optimization of the laser-arc hybrid welding process [3,4]. The D-optimal design of the second-order response model is also one of the RSM models [5,6,7]. The corresponding D-optimal design also includes three parts, namely, CCD (cube design, axial design and center point), but with different weights. It can be regarded as a differential weighted fractional factorial design. Ragavendrana et al. used the CCD model in the RSM method to design and optimize the process parameters of laser-TIG hybrid welding [8]. Ahn et al. selected the RSM method to optimize the process parameters of laser welding of Ti–6Al–4V [9]. Olabi et al. used the RSM to find optimal laser welding process parameters [10]. NAIT Salah et al. used the RSM method to analyze the factors affecting the quality of dissimilar alloy friction stir welding [11]. M. Subramanian et al. optimized the process parameters of friction stir welding by the RSM method [12]. Mirza et al. used face-centered central composite design (FCC) to optimize the process parameters of plasma arc welding of high strength low-alloy (HSLA) steel [13]. Cai et al. developed bead sidewall penetration using response surface method (RSM) based on central composite design (CCD), and numerically optimized narrow gap GMA welding [14]. J Ning developed an analytical model to study the temperature distribution in the metal powder bed additive manufacturing through analytical modeling. Considering the scanning strategy, the analytical solutions for the temperature prediction of single-track scan and multi-track scans are given [15]. The Taguchi strategy is also one of the factorial analysis methods. Its advantage is in finding the best design to minimize the expected loss (or mean square deviation) in uncontrollable noise space [16]. Juang adopted a modified Taguchi method to analyze the effect of each welding process parameter (arc gap, flow rate, welding current, and speed) on the weld pool geometry [17]. Lee et al. used the Taguchi method and regression analysis in order to optimize Nd-YAG laser welding parameters (nozzle type, rotating speed, title angle, focal position, pumping voltage, pulse frequency and pulse width) to seal an iodine-125 radioisotope seed into a titanium capsule [18]. The Taguchi method has been successfully applied to a number of industrial processes; including the automotive industry [19,20], robotics processing [21,22], plastics industries [23], and computer-aided design/electrical engineering tasks [24,25]. J Ning proposed an iterative gradient search method, based on the Kalman filter algorithm, to inverse Johnson-Cook model constant (J-C constant). The identified J-C constant is used to predict the machining force under different cutting conditions. This method has less experimental complexity and higher computational efficiency [26].

Response surface program involves experimental strategies, mathematical methods and statistical inference, so that users can effectively explore the system of interest [27]. RSM significantly reduces the number of experiments required for process parameter evaluation, analysis and optimization. Fitting the second-order model to the response variables of interest is an important aspect of RSM [16].

Differing from single heat source welding, laser-arc hybrid welding has many parameters, which affect each other and have complex variation rules, and this can bring inconvenience to experimental work. RSM was used to optimize process parameters, improve work efficiency and simplify research work. Compared with the existing research, the present work adopted the factorial experimental design method, used the analysis of variance and regression analysis to analyze the correlation of process parameters, and obtained the best process parameters. In addition, the purpose of this study also included evaluating the difference between the fitting of relevant experimental data, obtained by empirical model and model optimization, and optimizing the output signal-to-noise ratio.

In this paper, the BBD model of RSM was used to optimize the process parameters of laser-MAG hybrid welding of ship steel. The I-groove butt joint of ship steel plate was selected to simulate the backing welding in the production. The laser power, welding speed and welding current were taken as the response factors, and the weld penetration, tensile strength and impact absorption energy were taken as the response indices.

## 2. Materials and Methods

The base metal was 10CrNi3MoV marine steel, and the ex-factory state was quenched and tempered. The welding wire was WM960s with a diameter of 1.2 mm, and the shielding gas was 80% Ar + 20% CO_2_.

Table 1 shows the chemical composition of the base metal and welding wire used. Table 2 shows the mechanical properties of the base metal and welding wire.

**Figure 2:**
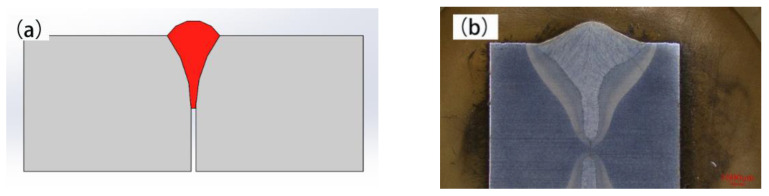
Welding diagram of I-groove joint (**a**) Welding diagram (**b**) Macroscopic.

The dimension of the base metal was 200 mm × 100 mm × 16 mm. I-groove single side butt welding was adopted. Both sides of the groove were cleaned before welding to remove oil stains. The welding diagram is shown in Figure 2a. Figure 2b is the macroscopic photo of the joint. The welding gap should not exceed 0.5 mm.

**Figure 3:**
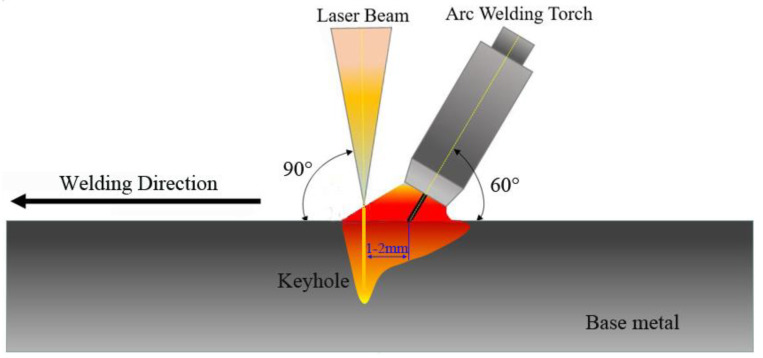
The schematic of laser-MIG hybrid welding system configurations.

IPG-4000 fiber laser and Kemppi MIG welding power were selected for the hybrid welding experiment. Kemppi MIG welding power was controlled by an expert system (welding current changes with wire feeding speed, and its value is controlled by expert system), with laser power (P), welding speed (V) and wire feeding speed (Vs) as variable factors, Weld penetration (DOP), tensile strength (Rm) and impact energy (Akv) were variables, and other process parameters, such as defocusing distance, and laser-wire distance were constant. The BBD model in design expert software was used for the experimental design. Table 3 shows the constant parameters of the laser-MAG hybrid welding process, and Table 4 shows the response design level value and coding value. The specific heat source configuration is shown in Figure 3.

**Figure 4:**
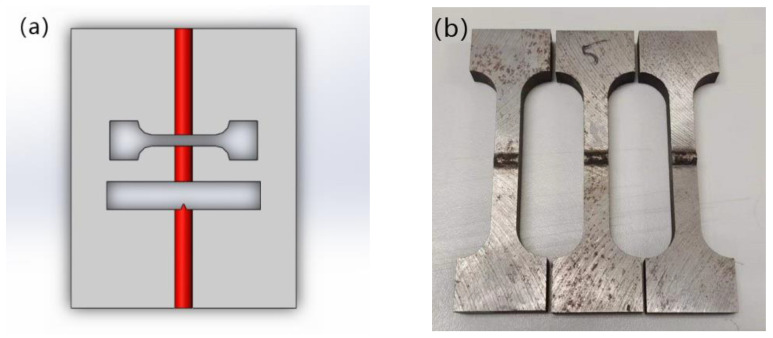

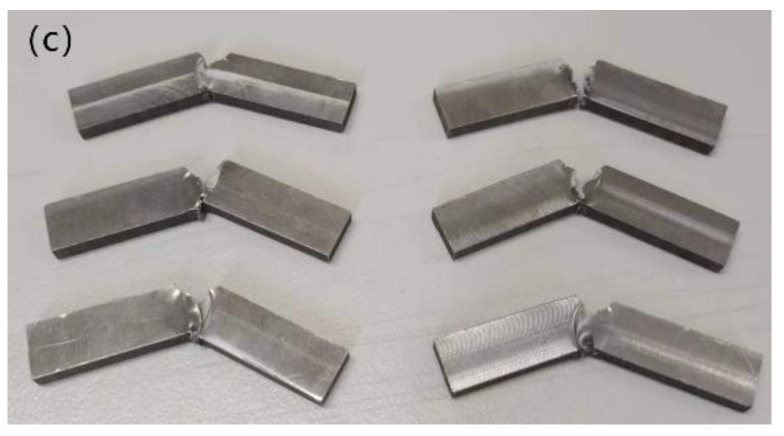
Schematic diagram of tensile and impact sampling (**a**) Sampling location; (**b**) Tensile test specimen; (**c**) Impact specimen.

Experiments were carried out according to the design matrix output by the software. The welding section was cut to measure and the penetration dimension recorded. Tensile and impact samples according to the corresponding penetration were tested. Refer to GB/T 228-2002 and GB/T 2650-2008 for the processing size, and Figure 4a shows the sampling diagram. Figure 4b,c show the appearance of the tensile and impact test specimen.

The test data was inputted into the design expert software for analysis. Table 5 shows the measured values of the design matrix.

## 3. Model Verification and Optimal Parameter Solution

### 3.1. Fitting Model Check and Model Optimization

Design Expert software was used for data analysis, and the results of variance (ANOVA) were verified according to the F test and the Lake of Fit test to obtain the best model.

#### 3.1.1. Test of Weld Penetration Fitting Model

Variance analysis was carried out for the weld penetration fitting model, and the results are shown in Table 6.

According to the F test, when the ‘*p*-value’ is less than 0.05, it is generally considered that the significance of the model is good and the accuracy of the model is high [28].

It can be seen from Table 5 that the ‘*p*-value’ of the penetration model was less than 0.0001, and the significance of the model was good. The smaller the ‘*p*-value’ of the response factor, the greater the influence of the process factors on the response index. The ‘*p*-value’ of A-P in the penetration model was 0.0113, and the ‘*p*-value’ of B-V and C-V were less than 0.0001, which indicated that the laser power, welding speed and wire feeding speed had a higher influence on the weld penetration, and the influence of welding speed and wire feeding speed on the penetration was higher than that of laser power.

Adeq Precision greater than 4 and C.V.% less than 10% indicated that the fitting model had strong resolution and high matching degree; R-squared was 0.8967, indicating that the model could explain 89.67% of the response value. R^2^ Adj-R^2^ Pred was less than 0.2, which indicated that the model could fully reflect the process of laser-MAG hybrid welding.

**Figure 5:**
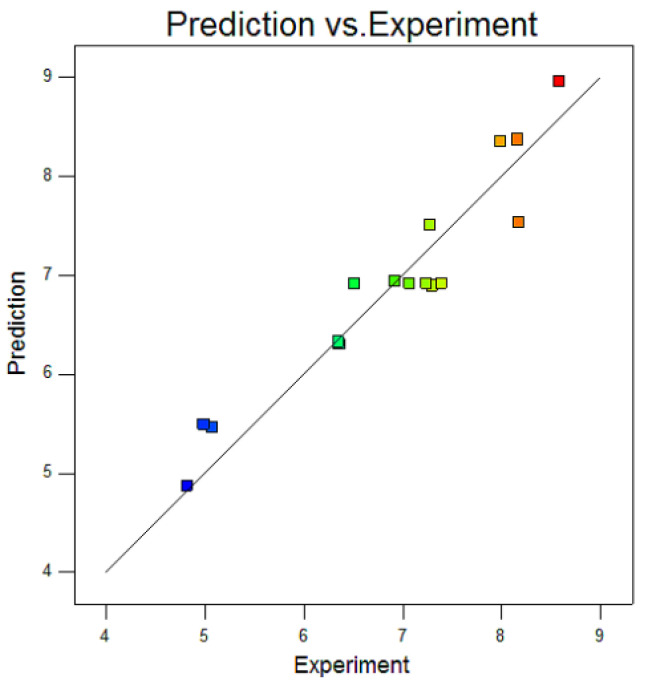
Comparison between predicted penetration of model and actual penetration value.

The predicted penetration value of the model was compared with the actual penetration value, and the results are shown in Figure 5. The predicted value of weld penetration was close to the actual value, which indicated that the model fitting of weld penetration was accurate.

Fitting model equation of weld penetration calculated by Design Expert:

Weld Penetration=3.80358+0.00192∗A−3.44583∗B+0.40350∗C

In order to determine the interactive influence of welding parameters on weld penetration, it was decided to analyze at the center point of response level (P = 3350 W, V = 0.9 m/min, vs. = 5.5 m/min).

**Figure 6:**
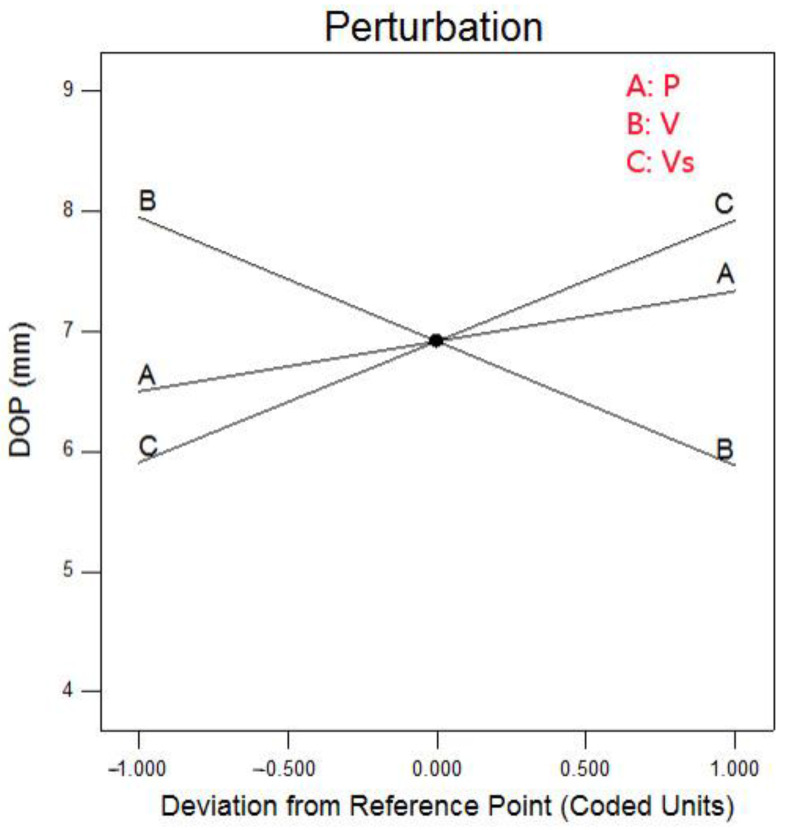
Influence of welding parameters on penetration.

In the BBD experiment design, the dimensionless linearization of each factor makes the coefficients of each order of the regression equation of the coded value irrelevant. In this way, the influence degree of the factors on the response value can be directly compared according to the absolute value of each coefficient in the regression equation. Figure 6 shows the influence of the welding parameters on the penetration. It can be seen that the influence of the parameters of laser-MAG hybrid welding on the penetration was linear. The influence order of welding parameters on penetration was V ≈ vs. > Power.

**Figure 7:**
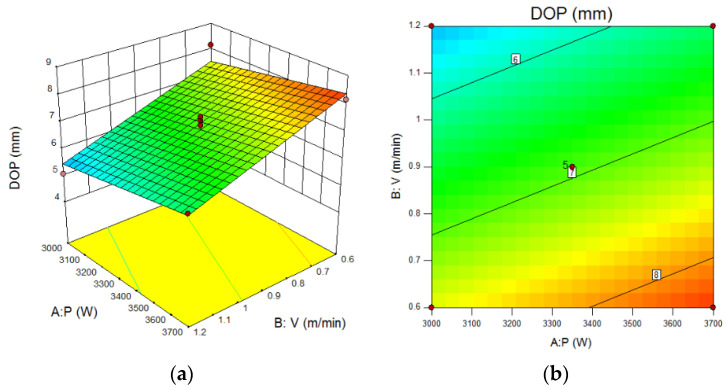

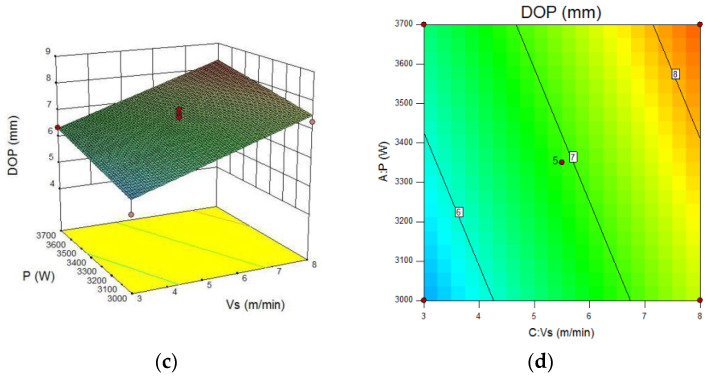
Effect of laser-MAG hybrid welding parameters on weld penetration (**a**) Contour map of P-V, (**b**) Isoline diagram of P-V, (**c**) Contour map of P-Vs, (**d**) Isoline diagram of P-Vs.

Figure 7 is the response contour map and isoline diagram of the influence of parameters on weld penetration.

Figure 7a,c show that the increase of laser power and wire feeding speed would increase the penetration, and the increase of welding speed would reduce the penetration. Figure 7b,d show that the change of laser power had little effect on the penetration, while the effect of the change of wire feeding speed and welding speed was more significant on the penetration.

The increase of laser power and welding current could not only improve the linear energy, but also improve the coupling effect and increase the penetration. The increase of welding speed reduced the linear energy, arc stability and penetration ability.

#### 3.1.2. Test of Tensile Strength Fitting Model

Variance analysis was carried out for the tensile strength fitting model, and the results are shown in Table 7.

In the analysis of variance of tensile strength, *p*-Value = 0.0181 < 0.05, R-squared = 0.8747, C.V.% = 1.34% < 10% and Adeq Precision > 4. It can be seen that the fitting equation of tensile strength had high significance and model accuracy, it could explain 87.47% of the response value, and the model had high resolution and matching degree.

**Figure 8:**
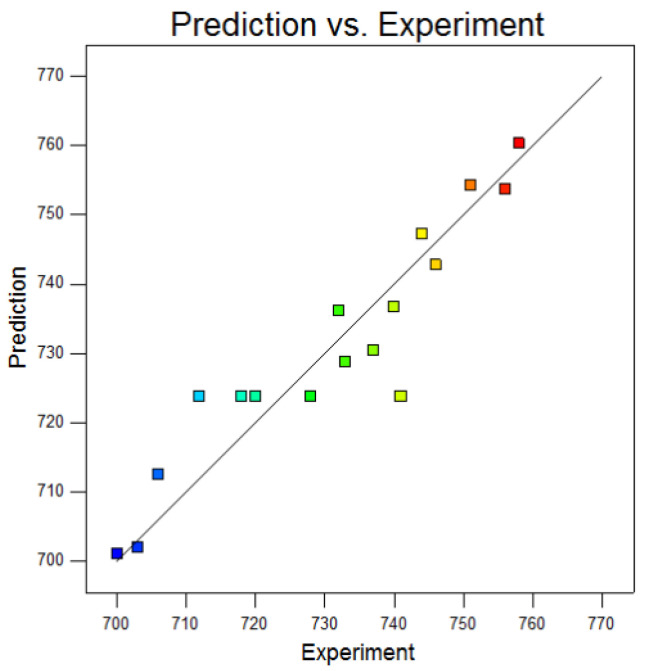
Comparison results between predicted strength of model and actual value.

The comparison results between the predicted strength value of the model and the actual strength value are shown in Figure 8. It can be seen that the accuracy of the tensile strength model was high.

The fitting model equation of tensile strength calculated by design expert is:

$$\begin{array}{ll} \mathrm{Tensile}\;\mathrm{strength} & =870.04552+0.078968*A-369.46429*B-30.96300*C-0.071429*A*B\\ & +\;0.011400*A*C+10.66667\mathrm{BC}\;-\;0.000013\;{*\;A}^{2}+303.75\;{*\;B}^{2}-1.702\;{*\;C}^{2} \end{array}$$

**Figure 9:**
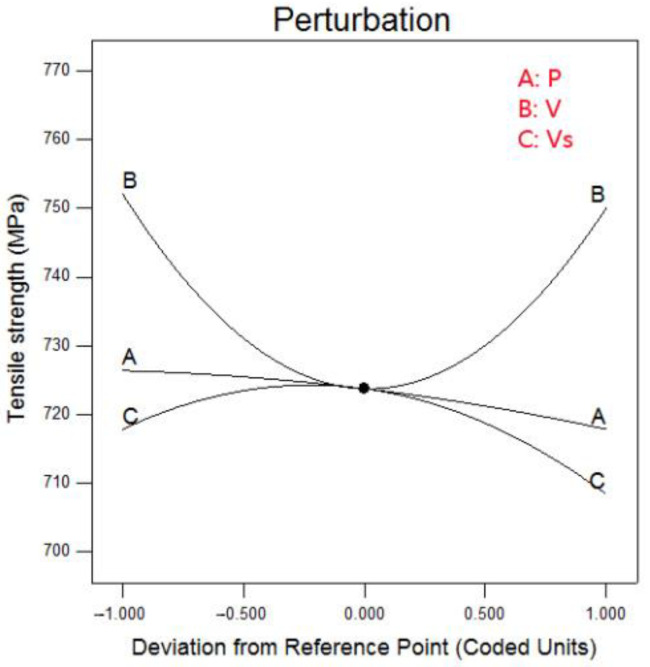
Effect of welding parameters on tensile strength.

**Figure 10:**
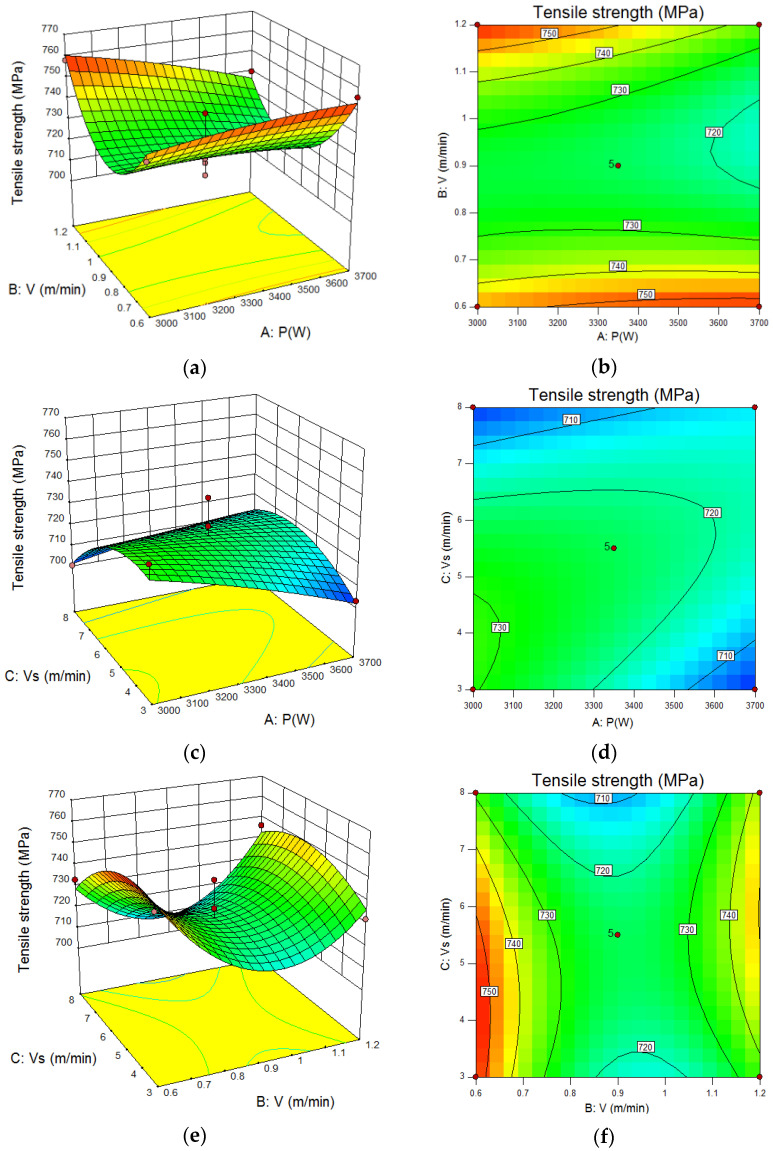
Contour map and isoline diagram of the effect of welding process parameters on tensile strength (**a**) Contour map of P-V, (**b**) Isoline diagram of P-V, (**c**) Contour map of P-Vs, (**d**) Isoline diagram of P-Vs, (**e**) Contour map of V-Vs, (**f**) Isoline diagram of V-Vs.

Figure 9 is a perturbation diagram of the effect of welding parameters on tensile strength. Figure 10 is the response contour map and isoline diagram of the influence of welding parameters on tensile strength.

It can be seen from Figure 10 that the laser power had little effect on the tensile strength, and the influence of welding speed on the tensile strength was higher than that of wire feeding speed.

In Figure 10, the response contour map presents a ‘U’ shape as a whole, and the contour map presents an ‘oval’ distribution, indicating that there are extreme points in it, which is conducive to determining the optimal parameter range.

Figure 10a,b show the interactive influence and isoline diagram of laser power and welding speed on tensile strength. It can be seen that the change of laser power had little influence on tensile strength. When the welding speed was low, increasing the laser power could slightly improve the tensile strength. When the welding speed was high, increasing the laser power would reduce the tensile strength. When the welding speed was 0.9 m/min, the tensile strength reached the lowest region of the cloud diagram, about 720 MPa.

Figure 10c,d show the interactive influence and isoline diagram of laser power and wire feeding speed on tensile strength. With the increase of wire feeding speed, the tensile strength increased and then decreased. The highest tensile strength appeared at low laser power and low wire feeding speed, more than 730 MPa.

Figure 10e,f show the interactive influence and isoline diagram of welding speed and wire feeding speed on tensile strength. It can be seen that the change of welding speed and wire feeding speed had a great influence on the tensile strength, and the response contour map showed a ‘saddle shape’. The tensile strength decreased with the increase of wire feeding speed. With the increase of welding speed, the influence trend of wire feeding speed on tensile strength remained unchanged, but the influence range became smaller. It reached the extreme point of the cloud map in the central point area, which was about 720 MPa.

#### 3.1.3. Test of Impact Absorption Energy Fitting Model

The variance analysis of the impact absorption energy model was carried out, and the results are shown in Table 8.

The *p*-value = 0.1878 > 0.05. In Table 7, it shows that the significance of the fitting equation of impact absorption energy was low, and the resolution and accuracy were low.

**Figure 11:**
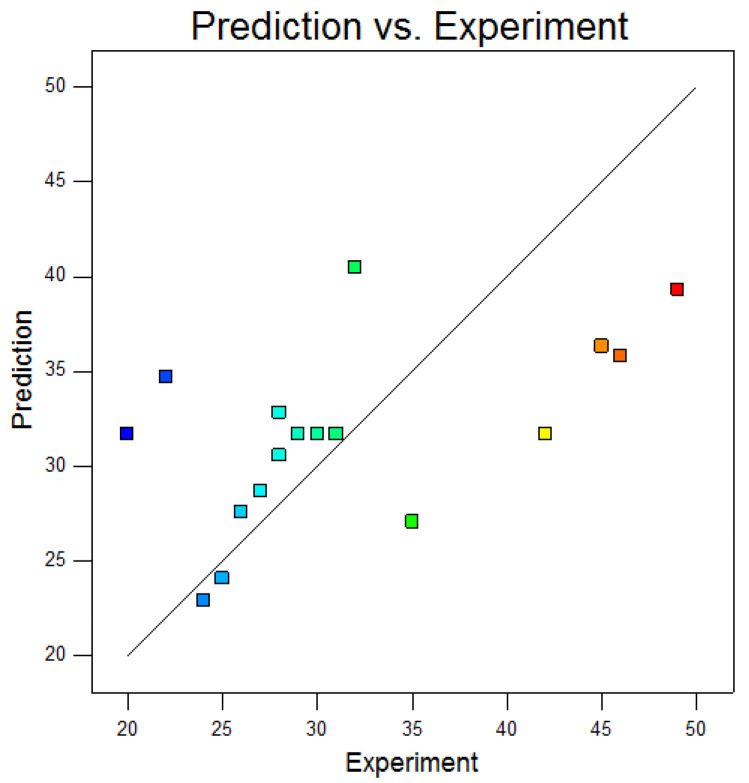
Comparison results between predicted impact absorption energy of model and actual value.

The comparison results between the predicted absorption energy and the actual value are shown in Figure 11. It can be seen that the accuracy of the comparison results was low. Combined with the test results, the analysis shows that the prediction accuracy of the impact absorption energy was too low, and the impact energy fitting model was not taken as the discussion category in the follow-up. When the model accuracy was verified by experiments after determining the optimal welding parameters, the impact energy results were tested and compared with the actual standards to ensure that the results of the selected process parameters met the acceptance standards.

The fitting model equation of impact absorption energy calculated by Design Expert:

Impact absorption energy=−8.99412+0.005∗A+19.58333∗B+1.15∗C

### 3.2. RSM Optimization Based on Expected Value Method

The model can optimize the parameters of the welding. The model optimization scheme is shown in Table 9, and the optimization results are shown in Table 10.

### 3.3. Verify the Optimal Solution of the Model

According to the optimal solution of the model, set the welding parameters for the experiment, and measure the penetration, tensile strength and impact absorption energy. The welding parameters are shown in Table 11, and the comparison results between the predicted value and the actual value are shown in Table 12.

According to the experiment results, it can be seen that the error between the predicted value of the model and the actual value was low, indicating that the accuracy of the optimized prediction model was high. The welding process scheme according to the RSM can provide more accurate guidance for actual production.

## 4. Conclusions

1. Based on the current situation of ship construction welding process, RSM experimental design method was used to optimize the process parameters of laser-arc hybrid welding, the groove size was optimized, and the process and experimental efficiency were greatly improved. At the same time, the performance of the welded joint was related to process parameters. Through the factorial experimental design of welding parameters and performance indices, the welding parameters were optimized.
2. RSM experimental design method identified the important influence of process parameters (laser power, welding speed and wire feeding speed) on weld penetration and mechanical properties of laser-arc hybrid welding, among which welding speed has the most significance.
3. Optimal welding parameters: P = 3700 W, V = 0.8 m/min, Vs = 7 m/min. On the premise that the mechanical properties meet the inspection standards, the maximum penetration can reach nearly 8 mm.

## Figures and Tables

**Table 1:** Chemical composition of base metal and welding wire (mass fraction, %).

Brand	C	Mn	Ni	Cr	Mo	Si	Ti	Cu	P	S	V
10CrNi3MoV	0.027	0.44	2.73	1.01	0.250	0.230	-	-	0.005	0.001	0.07
WM960S	0.041	1.38	2.80	-	0.268	0.495	0.044	0.079	0.008	0.005	0.10

**Table 2:** Mechanical properties of base metal and welding wire.

Brand	Rp_0.2_ (MPa)	Rm (MPa)	A (%)	Z (%)
10CrNi3MoV	670	730	21.6	72.2
WM960S	672	729	23.5	73

**Table 3:** Constant parameters of the laser-MAG hybrid welding process.

Constant Welding Parameters	Value
Defocus Distance	−2 mm
Laser-Wire Distance	1–2 mm
Extension Length of Welding Wire	15 mm
Shielding Gas Flow	20–25 L/min
Deflection Angle of Welding Torch	30°

**Table 4:** Response design level value and coding value.

Welding Parameters	Unit	−1	0	−1
P	W	3000	3350	3700
V	m/min	0.6	0.9	1.2
V_s_	m/min	3	5.5	8

**Table 5:** Experimental design matrix and the measured responses.

Run	Factor 1 A:P (W)	Factor 2 B:V (m/min)	Factor 3 C:Vs (m/min)	Response 1 DOP (mm)	Response 2 Rm (MPa)	Response 3 Akv (J)
1	3000	0.9	8	7.28	700	28
2	3350	0.9	5.5	7.07	728	42
3	3350	1.2	8	7.30	746	32
4	3000	0.6	5.5	8.17	744	25
5	3350	0.6	8	8.58	733	27
6	3350	0.9	5.5	6.51	712	31
7	3350	0.6	3	6.92	751	24
8	3350	0.9	5.5	7.39	741	20
9	3350	1.2	3	4.82	732	22
10	3700	0.9	3	6.35	703	28
11	3000	0.9	3	4.99	737	35
12	3700	1.2	5.5	6.36	740	49
13	3350	0.9	5.5	7.24	718	29
14	3350	0.9	5.5	7.39	720	30
15	3700	0.9	8	7.99	706	45
16	3000	1.2	5.5	5.08	758	46
17	3700	0.6	5.5	8.16	756	26

**Table 6:** Variance analysis of weld penetration fitting model.

Source	Sum of Squares	df	Mean Square	F Value	*p*-Value Prob > F	Source
Model	18.08	3	6.03	37.60	<0.0001	significant
A-P	1.39	1	1.39	8.70	0.0113	significant
B-V	8.55	1	8.55	53.33	<0.0001	significant
C-Vs	8.14	1	8.14	50.78	<0.0001	significant
Residual	2.08	13	0.16			
Lack of Fit	1.55	9	0.17	1.29	0.4326	Not significant
Pure Error	0.53	4	0.13			
Cor Total	20.17	16				
C.V. %		5.79		Adeq Precision		21.033
R-Squared		0.8967		R^2^ Adj-R^2^ Pred		0.09

**Table 7:** Variance analysis of tensile strength fitting model.

Source	Sum of Squares	df	Mean Square	F Value	*p*-Value Prob > F	Source
Model	4718.00	9	524.22	5.43	0.0181	significant
A-P	145.35	1	145.35	1.51	0.2594	
B-V	8.00	1	8.00	0.083	0.7818	
C-Vs	179.55	1	179.55	1.86	0.2148	
AB	225.00	1	225.00	2.33	0.1706	
AC	398.00	1	398.00	4.12	0.0818	
BC	256.00	1	256.00	2.65	0.1474	
A^2^	11.29	1	11.29	0.12	0.7424	
B^2^	3146.69	1	3146.69	32.60	0.0007	
C^2^	476.45	1	476.45	4.94	0.0617	
Residual	675.60	7	96.51			
Lack of Fit	174.80	3	58.27	0.47	0.7221	Not significant
Pure Error	500.80	4	125.20			
Cor Total	5393.60	16				
C.V. %		1.34%		R-Squared		0.8747
Adeq Precision		7.855		R^2^ Adj-R^2^ Pred		0.39

**Table 8:** Variance analysis of impact absorption energy model.

Source	Sum of Squares	df	Mean Square	F Value	*p*-Value Prob > F	Source
Model	366.75	3	122.25	1.85	0.1878	Not significant
A-P	24.50	1	24.50	0.37	0.5530	
B-V	276.13	1	276.13	4.18	0.0617	
C-Vs	66.13	1	66.13	1.0	0.3353	
Residual	858.78	13	66.06			
Lack of Fit	613.58	9	68.18	1.11	0.4967	Not significant
Pure Error	245.20	4	61.30			
Cor Total	1225.53	16				
C.V.%		25.63%		Adeq Precision		4.439
R-Squared		0.2993		R^2^ Adj-R^2^ Pred		0.05

**Table 9:** The model optimization scheme of impact absorption energy.

Process Parameters	Goal	Lower Limit	Upper Limit
P (W)	In range	3000	3700
V (m/min)	maximize	0.6	0.8
Vs (m/min)	In range	3	8
DOP	maximize	5	8.5
Tensile strength (MPa)	maximize	700	750
Akv (J)	maximize	20	49

**Table 10:** Model optimization results.

Number	P (W)	V (m/min)	Vs (m/min)	DOP	Rm (MPa)	AKv (J)	Desirability
1	3699.999	0.800	7.058	8.309	721.240	33.290	0.628
2	3700.000	0.800	7.036	8.300	721.333	33.264	0.628
3	3700.000	0.800	7.083	8.319	721.133	33.318	0.628
4	3699.996	0.800	7.111	8.330	721.010	33.350	0.628

**Table 11:** Welding process parameters.

P (W)	V (m/min)	Vs (m/min)	Defocus Amount (mm)	Laser-Wire Distance (mm)	Shield Gas Flow (L/min)
3700	0.8	7	−2	2	20

**Table 12:** Comparison results between model predicted value and actual value.

Target	Predicted Result	Actua Result	Error
DOP	8.3 mm	7.8 mm	5%
Strength	721 MPa	723 MPa	0.2%
Akv	33 J	34 J	3%

## Data Availability

Not applicable.
